# Advancing Immunotherapy in Pancreatic Cancer: A Brief Review of Emerging Adoptive Cell Therapies

**DOI:** 10.3390/cancers17040589

**Published:** 2025-02-09

**Authors:** Deepak Sherpally, Ashish Manne

**Affiliations:** 1 Department of Internal Medicine, New York Medical College, Metropolitan, New York, NY 10029, USA; 2 Department of Internal Medicine, Division of Medical Oncology, The Arthur G. James Cancer Hospital and Richard J. Solove Research Institute, The Ohio State University Comprehensive Cancer Center, Columbus, OH 43210, USA; ashish.manne@osumc.edu

**Keywords:** pancreatic ductal adenocarcinoma, adoptive cell therapy, chimeric antigen receptor T cells (CAR-T), chimeric antigen receptor NK cells (CAR-NK), tumor-infiltrating lymphocytes (TIL), T-cell receptor (TCR)-engineered T cells, cytokine-induced killer cells (CIK)

## Abstract

Pancreatic cancer has the lowest 5-year survival rate (13%) among major cancers and is the third leading cause of cancer-related deaths in the United States. The high lethality of this cancer is attributed to its insidious onset, late-stage diagnosis, rapid progression, and limited treatment options. Addressing these challenges requires a deeper understanding of the complex tumor microenvironment to identify novel therapeutic targets. Newer approaches like adoptive cell therapy have shown remarkable success in treating hematological malignancies, but their application in solid tumors, particularly pancreatic cancer, is still in the early stages of development. ACT broadly involves isolating immune cells (T lymphocytes, Natural Killer cells, and macrophages) from the patient, followed by genetic engineering to enhance and mount a specific anti-tumor response. Various ACT modalities are under investigation for pancreatic cancer, including chimeric antigen receptor T cells (CAR-T), chimeric antigen receptor NK cells (CAR-NK), tumor-infiltrating lymphocytes (TIL), T-cell receptor (TCR)-engineered T cells, and cytokine-induced killer cells (CIK). Major hurdles have been identifying actionable tumor antigens and delivering focused cellular therapies to overcome the immunosuppressive and dense fibrotic stroma surrounding the pancreatic cancer. Further studies are needed to explore the limitations faced by cellular therapy in pancreatic cancer and identify novel combination treatment approaches in order to improve clinical outcomes.

## Simple Summary

Pancreatic cancer is an aggressive and highly lethal malignancy with limited treatment options as it is usually detected in the advanced stage. There is an urgent need to explore new therapeutic options, and recent studies on adoptive cellular therapy (ACT) look promising, although they are in the early stages. We attempted to review the completed and ongoing studies on ACT to explore the current and future approaches to pancreatic cancer management.

## 1. Introduction

Pancreatic cancer is among the most lethal malignancies, representing a significant global health challenge. In 2024, it is projected to cause approximately 51,000 deaths out of 66,000 newly diagnosed cases in the United States alone [1]. With a 5-year survival rate of just 13%, pancreatic cancer has the lowest survival rate among the major cancers. Its incidence is similar among African-American and Caucasian populations. Despite accounting for only 3% of new cancer diagnoses in the United States, pancreatic cancer is currently the third leading cause of cancer-related deaths and is expected to become the second in the near future [2]. Globally, the European region exhibits the highest age-standardized incidence and mortality rates, while the Southeast Asia region reports the lowest [3]. The high lethality of pancreatic cancer is attributed to its insidious onset, late-stage diagnosis, aggressive progression, and limited treatment options. Addressing these challenges requires a deeper understanding of the tumor microenvironment (TME) to identify novel therapeutic targets and expand treatment options, ultimately improving patient outcomes in the long term.

Pancreatic cancer risk factors are broadly categorized into modifiable and non-modifiable factors [4]. Key modifiable risk factors include smoking, excessive alcohol consumption, diets high in red or processed meats, obesity, and infections such as Helicobacter pylori. These factors contribute to the higher incidence observed in developed countries. Non-modifiable risk factors include advanced age, male gender, ethnicity, specific blood groups, microbiota composition, genetic predisposition, and diabetes mellitus. A comprehensive understanding of these risk factors is critical for developing effective prevention and intervention strategies.

## 2. Current Management of Pancreatic Ductal Adenocarcinoma

Pancreatic ductal adenocarcinomas (PDAC) account for approximately 90% of primary pancreatic cancers, with the remainder comprising less common types such as squamous, acinar, signet-ring (exocrine), neuroendocrine, and undifferentiated carcinomas [5]. PDAC is typically stratified for risk and management using the tumor-node-metastasis (TNM) system outlined in the eighth edition of the American Joint Committee on Cancer (AJCC) staging manual [6]. While TNM staging informs treatment and prognosis, it does not give sufficient information critical for surgical planning. Another widely used classification system focuses on tumor resectability and the presence of distant metastatic disease at diagnosis [7]. Based on this approach, PDAC is categorized as resectable (R), borderline resectable (BR), locally advanced (LA), or metastatic [8,9,10,11]. Resectability is determined by the degree of tumor involvement with surrounding arteries and veins, typically assessed in a multidisciplinary setting. In R-PDAC, there is no tumor contact with adjacent blood vessels. BR-PDAC involves some tumor contact with blood vessels, with the expectation that systemic chemotherapy (CT) or radiation therapy (RT) can convert these cases to R-PDAC. LA-PDAC, a less clearly defined category, includes tumors with significant involvement of major arteries (e.g., celiac trunk or superior mesenteric artery interface >180°) or veins, rendering both resection and vascular reconstruction infeasible. Following stratification, treatment plans are tailored to the disease stage. For R-PDAC and BR-PDAC, neoadjuvant chemotherapy (NAT), often combined with RT, is now preferred prior to surgical resection. In LA-PDAC, NAT helps to identify patients who may benefit from subsequent surgery. For metastatic PDAC and certain LA cases, clinical trial enrollment is recommended. Systemic chemotherapy, using regimens such as FOLFIRINOX or gemcitabine/nab-paclitaxel (G/NP), remains the cornerstone of treatment for advanced PDAC. Liposomal irinotecan combinations with 5-flurouracil (5FU) and oxaliplatin (NALIRIFOX) is another option for treating naïve PDA patients and is often used in combination with 5FU alone following disease progression on G/NP [12,13]. However, outcomes for metastatic PDAC remain poor, with a 5-year survival rate of only 3% [14,15].

## 3. Immunotherapy in PDAC

Immunotherapy in PDAC focuses on leveraging the TME and the host immune system [16]. Immune checkpoint inhibitors (ICIs), targeting cytotoxic T-lymphocyte-associated protein 4 (CTLA-4) and programmed cell death protein 1 (PD-1), have shown efficacy in mismatch repair-deficient (MMR-D) tumors, but this subgroup represents only 2% of PDAC patients [17,18,19]. While the ICIs have improved the outcomes of esophageal, liver, and biliary tract cancers, their success in mismatch repair-proficient PDAC remains limited [20,21,22,23,24,25,26,27,28,29]. Emerging immunotherapy strategies, such as oncolytic virus therapy (OVT), adoptive cell transfer therapy (ACT), and cancer vaccines—including innovative KRAS mutant peptide-based vaccines in adjuvant settings—show promising potential across various tumor types, including PDAC [23,30]. Among these, chimeric antigen receptor (CAR) T-cell therapy is being actively investigated as a novel therapeutic strategy in PDAC. The urgent need for new targets and treatment modalities highlights the potential of immunotherapy as a critical avenue for improving outcomes in this challenging and aggressive disease.

### 3.1. Adoptive Cell Therapy in PDAC

**Figure 1:**
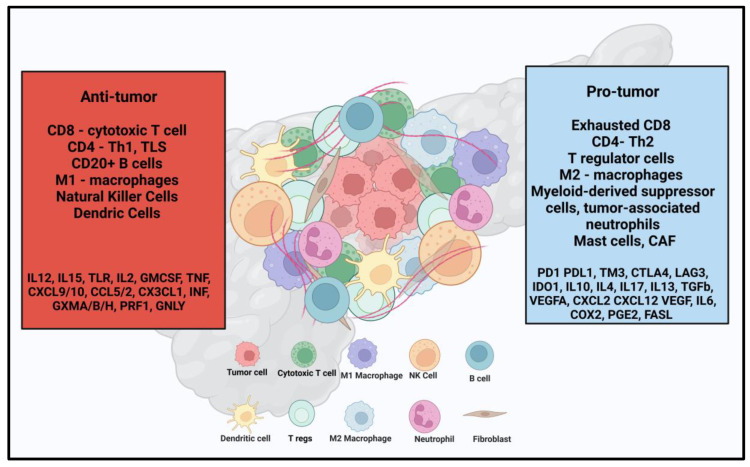
Summarizing the tumor microenvironment in pancreatic ductal adenocarcinoma. Tregs—regulatory T cells; NK—natural killer.

TME is a complex ecosystem that surrounds tumor cells, comprising various immune cell populations that play critical roles in maintaining its pro-tumorigenic nature [18]. These immune cells include lymphocytes (T and B cells), macrophages, natural killer (NK) cells, dendritic cells (DCs), myeloid-derived suppressor cells (MDSCs), neutrophils, and mast cells, as illustrated in Figure 1. Each cell type contributes uniquely to the dynamic interplay within the TME, promoting tumor growth, immune evasion, and resistance to therapy. The immunosuppressive role of tumor cells, tumor vasculature, cancer-associated fibroblasts (CAFs), and the microbiome in immune evasion is well-established [31]. CAFs contribute to immune resistance by secreting extracellular matrix components such as collagen, fibronectin, and hyaluronan, which create a dense physical barrier that restricts blood flow and limits T-cell infiltration into the TME [32,33]. Additionally, tumor endothelial cells and pericytes play a crucial role in neovascularization, promoting the formation of abnormal blood vessels around the tumor [34,35]. These cells release various immunosuppressive signals that hinder T-cell recruitment and impair their anti-tumor activity.

Immune-related TME is important not only for the effectiveness of ICI or other cellular therapy modalities but also for the outcomes, affecting both prognosis and the treatment response. High infiltration of anti-tumor immune cells significantly improved the outcomes of PDA, irrespective of ICI use [36,37,38,39,40,41,42,43].

There is a growing interest in targeting TME to treat PDAC, and ACT is emerging as a key strategy in this effort [23,44]. While ACT has shown remarkable success in treating hematological malignancies, its application in solid tumors, including PDAC, remains in the early stages of development [45,46]. ACT broadly involves isolating immune cells—such as T lymphocytes, NK cells, and macrophages—from the patient, followed by their re-engineering and genetic modification to enhance their anti-tumor activity [47]. Various ACT modalities are under investigation for PDAC, including chimeric antigen receptor T cells (CAR-T), chimeric antigen receptor NK cells (CAR-NK), tumor-infiltrating lymphocytes (TILs), T-cell receptor (TCR)-engineered T cells, and cytokine-induced killer cells (CIK cells). These approaches are currently being evaluated in clinical trials to improve outcomes for this challenging malignancy. The following sections will delve deeper into these ACT modalities and their potential impact on PDAC treatment.

#### 3.1.1. CAR-T in PDAC

The CAR-T cell is a form of ACT that redirects a patient’s T cells to specifically target cancer cells through genetic engineering [48]. CARs are synthetic receptors designed with four main components: an extracellular antigen-binding domain, a hinge region, a transmembrane domain, and one or more intracellular signaling domains [46]. Since the development of first-generation CARs in 1989, subsequent generations have undergone significant advancements to enhance clinical efficacy [49]. To date, five generations of CAR-T cells have been developed, each featuring modifications to the domain structure and the inclusion of additional co-stimulatory molecules. Newer generations of CAR-T cells demonstrate improved T-cell activation, enhanced efficacy, and greater persistence, with the ability to rapidly expand and survive long after infusion.

**Figure 2:**
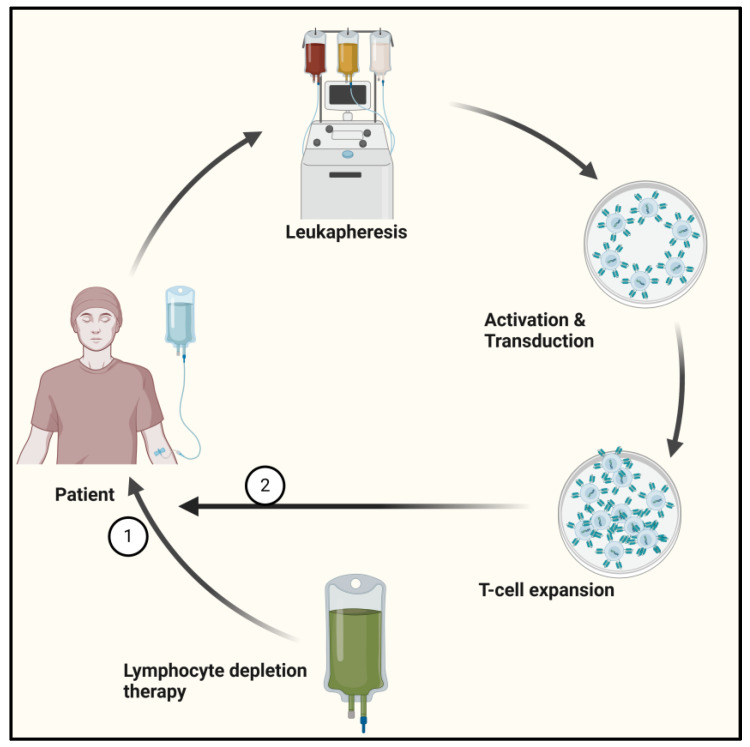
CAR-T cell manufacturing. 1/2—Lymphocyte depletion chemotherapy is given before CAR-T cell infusion.

CAR-T cell production is a complex, multi-step process (see Figure 2) that can be summarized as follows: it starts with leukapheresis to isolate the lymphocytes from the patient (autologous) or donor (allogeneic), followed by their stimulation through antibody-coated beads or plate-bound antibodies to activate them, leukocytes are then transduced with the CAR gene using lentiviral vectors or gamma-retroviral vectors to express the CAR molecule, lymphocytes are expanded in culture to a sufficient number required for the treatment, and CAR-T cells are injected into the patient to simulate the in vivo conditions and lymphodepletion chemotherapy after appropriate quality checks [50,51]. Fresh CAR-T cells should ideally be administered within 24 h of preparation; however, they are often cryopreserved at −180 °C for transportation and can be stored for 10–14 days.

We summarized the results of completed early-phase clinical trials (Phase I or I/II) in Table 1 and ongoing trials in Table 2.

The principal toxicities associated with CAR-T cell therapy are cytokine release syndrome (CRS) and immune effector cell-associated neurotoxicity syndrome (ICANS) [72]. CRS presents clinically with a spectrum of symptoms, ranging from mild flu-like manifestations to severe vasodilatory shock and end-organ dysfunction, potentially leading to life-threatening complications. Management of CRS involves supportive care, including symptomatic treatment and the use of tocilizumab, an interleukin-6 (IL-6) receptor antagonist, with or without corticosteroids, depending on the severity of the condition. ICANS typically develops after the onset of CRS and exhibits a range of neurological symptoms, from temporary cognitive deficits to fatal cerebral edema. The management of ICANS is stratified by severity: mild cases are treated with supportive measures, severe cases with corticosteroids, and anti-IL-6 therapy is employed only if ICANS occurs alongside CRS. The underlying factors contributing to these toxicities include antigen overlap between cancerous and normal tissues, leading to off-target effects and an exaggerated immune response triggered by CAR-T cell activation.

#### 3.1.2. Tumor-Infiltrating Lymphocyte Therapy (TIL) in PDAC

TIL-based ACT involves isolating TILs from tumor tissues, expanding them in vitro, and reinfusing them into patients to identify and destroy tumor cells [73]. TIL therapy, which has shown promising results in solid tumors such as melanoma, breast, and ovarian cancers, is now being investigated for PDAC [74,75,76,77,78,79,80,81,82,83,84,85,86,87]. In a meta-analysis that examined PDAC-TME, a higher CD8+ T-cell subgroup was associated with significant survival benefits, highlighting the potential of TIL therapy in PDAC [38]. TILs therapy has unique advantages, including its ability to target tumor-specific neoantigens due to the presence of multiple T-cell receptor clones, its ease of extraction from tumor tissue owing to the high number of effector memory T cells, and its low toxicity profile since it utilizes autologous cells without genetic modification [73,88].

TILs production involves isolating naturally occurring lymphocytes from the tumor tissue obtained via biopsy or surgery [89]. These lymphocytes are expanded ex vivo and re-infused into the patient to combat cancer. TILs are cultured in high doses of interleukin-2 (IL-2) and undergo rapid expansion using anti-CD3 antibodies. Due to the complexity and labor-intensive nature of manufacturing, TIL therapy is available only at specialized cancer centers and requires several weeks for production. Efforts are underway to simplify this process by incorporating closed-system bioreactors and leveraging blood bank infrastructure to improve patient accessibility. If immediate infusion is not feasible, TILs can be cryopreserved and transported. After lymphodepletion chemotherapy (cyclophosphamide and fludarabine), TILs are infused, followed by a high-dose bolus of IL-2 every 8–12 h for 2–5 days (to enhance their persistence and function) [90].

In a study involving 17 patients, including 5 with PDAC, the best response was observed in a PDAC patient with stable disease for 17 months [88]. However, this patient, who had liver and peritoneal metastases, exhibited no response at the primary tumor site. Overall, no objective responses (OR) were recorded among the PDAC cohort, with three achieving stable disease (SD) and two progressing (PD). Progression-free survival (PFS) and overall survival (OS) for the PDAC patients were 2.43 months and 14.49 months, respectively, which were worse compared to the overall study population (PFS: 2.53 months, OS: 18.86 months) [70]. Bone marrow suppression emerged as a concerning high-grade adverse event across the entire study cohort. TIL therapy for PDAC remains in its early stages, and ongoing clinical trials are summarized in Table 3. Continued research is essential to optimize this approach and improve patient outcomes with PDAC.

#### 3.1.3. CAR-NK Cell Therapy

NK cells play a pivotal role as part of the body’s first line of defense against cancer. Genetically engineered NK cells have demonstrated the ability to mount specific and targeted anti-tumor responses, offering a promising avenue for cancer immunotherapy [91]. CAR-NK cell manufacturing is also complex, like CAR-T production, and involves the following steps: NK cells are isolated in sufficient numbers from peripheral blood mononuclear cells (PBMC) of a healthy donor using isolation kits; they are stimulated and engineered to express CARs and expanded with cytokines for clinical-grade application and activated CAR-NK cells are administered without irradiation allowing them to expand in vivo [92,93]. CAR-NK cells offer advantages over CAR-T cells, including lacking HLA matching requirements, reduced graft-versus-host disease (GVHD) risk, and off-the-shelf availability for multiple patients. These cells can be derived from sources such as PBMCs, umbilical cord blood (UCB), CD34+ hematopoietic progenitor cells (HPCs), induced pluripotent stem cells (iPSCs), and established cell lines (e.g., NK-92, NKG, NKL) [92]. NK cells exhibit anti-tumor responses through both CAR-dependent and CAR-independent mechanisms, such as antibody-dependent cellular cytotoxicity (ADCC).

Preclinical studies involving chimeric antigen receptor NK (CAR-NK) cells targeting prostate stem cell antigen (PSCA) and mesothelin in PDAC have shown encouraging results, raising hope for their clinical application in select patient populations [94,95]. Compared to CAR-T cells, CAR-NK cells offer potential advantages, including reduced toxicity due to their shorter half-life and distinct cytokine profile, as well as a lower likelihood of inducing alloreactivity, making them suitable for “off-the-shelf” therapeutic products [23,96]. However, several limitations hinder their clinical implementation. These include technical challenges in manufacturing, poor tumor infiltration, and the short half-life of NK cells, which necessitates repeated administrations to sustain therapeutic effects [97,98]. We discussed ongoing CAR-NK trials in Table 3.

#### 3.1.4. TCR-Engineered T-Cell Therapy

Genetically engineered T-cell receptor (TCR-T) therapy modifies TCRs to recognize and eliminate tumor cells [99,100]. Unlike CARs, which require tumor antigens to present on the cell surface, TCRs detect intracellular proteins presented via the human leukocyte antigen (HLA) system. TCR-T cells are generated by collecting T cells from either patients (autologous) or healthy donors (allogeneic) and stimulating them in vitro to selectively expand those with the desired specificity [101,102,103,104]. Stimulation is achieved using antigen-presenting cells (either autologous or artificial) that present the target antigen through exogenous peptide or cDNA/RNA delivery. Antigen-specific T cells are isolated using methods such as magnetic bead separation, fluorescence-activated cell sorting (FACS), or IFN-γ capture assays. The T cells of interest are then cloned via polymerase chain reaction (PCR) amplification using various techniques, including limiting dilution, single-cell reverse transcription PCR (RT-PCR), and single-cell RNA sequencing. Prior to infusion, patients undergo lymphodepletion therapy with cyclophosphamide and fludarabine to enhance the engraftment of the genetically engineered T cells. Although these cells can be cryopreserved for extended periods, their viability significantly declines within six hours of thawing [105].

Commonly targeted antigens in TCR therapy include mesothelin (MSLN), epidermal growth factor receptor (EGFR), claudin 18.2 (CLDN), CD133, and human epidermal growth factor receptor 2 (HER2). Notably, a study by Leidner et al. demonstrated that TCRs targeting mutant KRAS (KRAS12D) elicited responses in one patient with metastatic PDAC, highlighting the potential of TCR-based therapies in this challenging cancer type [106]. We discussed the ongoing TCR trials in Table 3.

#### 3.1.5. Cytokine-Induced Killer (CIK) Cells

CIK cells are a heterogeneous group of CD8+ T (CD3+CD56−) cells that exhibit a hybrid phenotype, combining features of both T cells and natural killer (NK) cells. These cells are generated by incubating human-derived peripheral lymphocytes with anti-CD3 antibodies and cytokines [107,108,109]. CIK cells have shown the potential to enhance the efficacy of other anti-cancer therapies, such as ICIs and chemotherapy, by amplifying anti-tumor responses [110]. It addresses several limitations associated with CAR-T therapy, including the need for large blood volumes for leukapheresis, complex gene transfer processes, and high production costs [111].

Preclinical and clinical studies have demonstrated synergistic effects when CIK therapy is combined with chemotherapy in pancreatic cancer [108,109]. In a randomized study evaluating the addition of the chemotherapy agent S-1 to CIK therapy, a slight improvement in progression-free survival (PFS) was observed (2.5 months vs. 2.9 months, *p* = 0.03), although overall survival (OS) was comparable between the groups (6.1 months vs. 6.6 months, *p* = 0.09) [109]. Hematological toxicity was similar across both groups, but the incidence of non-infectious fever was significantly higher in the CIK group (32% vs. 3.3%, *p* = 0.004). In another study involving 47 patients with advanced pancreatic ductal adenocarcinoma (PDAC), the median OS and PFS were notably higher in the group treated with dendritic cell-CIK (DC-CIK) therapy combined with S-1 (212 and 136 days, respectively) compared to those receiving DC-CIK therapy alone (128 and 85 days), chemotherapy alone (141 and 92 days), or supportive care only (52 and 43 days) [112]. These findings suggest that CIK-based therapies, particularly when combined with other modalities, hold promise for improving outcomes in advanced PDAC patients. We discussed the ongoing CIK cell therapy in PDAC in Table 3.

## 4. Discussion

ACTs have shown great promise in cancer treatment; however, their clinical success remains challenged by several obstacles. Despite these hurdles, ongoing research and innovation are continuously advancing strategies to enhance their effectiveness and overcome existing limitations.

### 4.1. Challenges in ACT

Immunosuppressive TME is an independent poor prognostic factor and presents a significant challenge to achieving effective outcomes with immune therapies, including ICI and ACT [31]. These challenges are not unique to PDAC but impact their broader clinical application. The success of CAR-T therapy largely depends on the selection of appropriate targets. Many current studies have repurposed tumor-associated antigens (TAAs) from other malignancies—such as CEA, Claudin, mesothelin, EGFR, and HER2—with varying degrees of success. The modest responses observed in past clinical trials, combined with a high risk of toxicities such as CRS and ICANS, emphasize the need to identify more reliable target antigens. Key concerns include antigen escape (loss of target antigen expression in tumor cells), off-target effects (attack on normal cells expressing the target antigen), and the potential for secondary malignancies [46,113]. CAR-T cell exhaustion, characterized by impaired T-cell proliferation and effector function due to persistent antigen stimulation, remains a major cause of treatment resistance and tumor relapse [114].

Similar challenges exist with TCR-T therapies, including target selection, exhaustion, toxicity, and off-target effects. Additionally, TCRs are restricted to recognizing antigens presented by HLA class I molecules, which may limit their applicability to tumors with heterogeneous antigen presentation [115]. Despite the advantages of CAR-NK therapy over CAR-T therapy, challenges such as difficulty in ex vivo expansion, freezing and storage limitations, lower cytotoxicity compared to CAR-T cells, low circulating levels, and limited commercial partnerships hinder its clinical adoption [116].

In contrast, TIL therapy offers the advantage of using autologous cells without extensive genetic modification, thereby reducing toxicity risks. However, several logistical and economic barriers hinder its widespread use, including the requirement for fresh tumor tissue, the labor-intensive process of generating and expanding TILs with IL-2, anti-CD3, and feeder cells, and the eventual reinfusion into the patient [117]. Furthermore, conditioning chemotherapy (cyclophosphamide and fludarabine) used prior to TIL infusion often results in bone marrow suppression, while IL-2 administration is associated with adverse effects such as tachycardia, hypotension, rash, diarrhea, dyspnea, anuria, and edema [118]. The heterogeneity of cell populations is the main drawback of CIK cellular therapies [119]. An increased presence of CD4+ T cells and regulatory T cells (Tregs) within the TME can impair cytotoxicity and induce T-cell exhaustion, ultimately reducing the treatment’s efficacy [120].

In conclusion, while immune therapies such as CAR-T, TCR-T, TIL, CAR-NK, and CIK therapies hold significant promise for the treatment of PDAC and other malignancies, several challenges must be addressed to optimize their efficacy and safety.

### 4.2. Future Directions in ACT

Enhancing the TME to reduce immunosuppression and improve T-cell infiltration is crucial for the success of ACTs. Strategies such as targeting angiogenesis, depleting tumor-associated macrophages, and inhibiting cancer-associated fibroblasts responsible for the dense extracellular matrix have shown promise in preclinical studies [121,122,123,124,125,126]. Therapeutic approaches involving vascular endothelial growth factor A (VEGFA) and angiopoietin-2 inhibitors, as well as other signaling pathways, are currently being explored in combination with ACTs [127,128,129,130].

Identifying novel PDAC-specific targets is crucial for advancing CAR-T and TCR therapies. Several promising targets currently under investigation for PDAC include natural killer group 2D (NKG2D), fibroblast activation protein (FAP), CD318, TSPAN8, and CD66c [131,132,133]. Innovative approaches such as TCR-CAR and TCR-like CAR, which combine the strengths of CAR and TCR platforms, are being explored to enhance therapeutic efficacy [134]. In TCR-CAR, a soluble TCR is fused to the CAR signaling tail, while TCR-like CAR incorporates TCR-like antibodies capable of recognizing the peptide/major histocompatibility complex (MHC) on tumor cell surfaces, combined with CAR signaling to improve target specificity [135,136]. Additionally, combining CAR-T therapy with PD-1 blockade—either via ICIs or intrinsic signaling pathway modifications—is being explored to mitigate CAR-T exhaustion and enhance efficacy [137,138,139,140,141,142,143]. Early-phase studies have demonstrated potential in prostate, breast, and pleural malignancies; however, similar efforts in PDAC remain limited. Likewise, the combination of CAR-T therapy with transforming growth factor beta (TGF-β) signal blockade is being explored to counteract the immunosuppressive TME [144,145,146,147]. Additionally, CAR-T therapy is being studied in combination with other agents, including CD20-targeting antibodies (rituximab, obinutuzumab, and glofitamab), Bruton tyrosine kinase (BTK) inhibitors (acalabrutinib and ibrutinib), and lenalidomide, to further enhance efficacy [148]. In prostate cancer, CAR-T is also being evaluated in conjunction with radiation therapy (NCT05805371), and vaccine combinations are currently under review for hematological malignancies [148]. Encouraging similar research efforts in PDAC is critical to expanding treatment options and improving patient outcomes.

Furthermore, various novel CAR designs are being developed to prevent prolonged antigen exposure and enhance control over CAR-T cell activity, such as drug-stabilizing, drug-destabilizing, inducible (via doxycycline), self-driving (under control of AP1-NF-kB or STAT5), TME-driven (induced by IFN-γ, NFκB, and hypoxia), switch, and split CARs [149,150,151,152,153]. The switch and split CARs are engineered by uncoupling the activation domains to prevent prolonged exposure to the target. Synthetic Notch (SynNotch) receptors and bispecific adapters, which drive the expression of CARs upon tumor antigen recognition, are in early development stages and show the potential to enhance therapeutic outcomes [154,155]. Synthetic TCR and antigen receptor (STAR) and T-cell receptor fusion constructs (TRuCs)-T cells integrate antigen-recognition domains to improve specificity and reduce the toxicity of TCR-T cells [156,157]. An antibody-based binding domain fused to TCR in TRuCs and a double-chain chimeric receptor are constructed that incorporates the antibody’s antigen-recognition domain and constant regions of TCR that engage endogenous CD3 signaling machinery in STARs.

TIL therapy continues to evolve, with combinations involving ICIs showing promise in cancers such as breast, head and neck, cervical, colon, thyroid, and melanoma [85,158,159,160,161,162]. Research is currently exploring next-generation TILs, including tumor-infiltrating B cells (TIL-B) and PD-1-inactivated TILs, to optimize their therapeutic efficacy, the development of modified interleukins, such as IL-2 superkines with enhanced affinity for the IL-2 β subunit and IL-15 super agonist complexes, is being investigated to enhance their clinical potential further [163,164,165]. Improvement in the processes involved in TILs therapies, such as automation of various stages (cell isolation and expansion) using the latest technology, standardization of the protocols, and strong collaborations among the stakeholders (industries, hospitals, and healthcare leaders) will be crucial in scaling up TIL therapy for widespread clinical use [166,167].

CAR-NK cells are also being evaluated in combination with ICIs and other agents, such as lenalidomide and azacytidine, to improve their efficacy [168]. Standardized protocols for cytokine-induced killer (CIK) cell expansion are needed to optimize cell composition (T regs and CD4 cells) and efficacy. Retrospective studies have shown promising results when combining CIK therapy with chemotherapy and ICIs, highlighting its potential in PDAC and other malignancies [169,170,171,172,173]. Gene editing remains a critical area of research, with clustered regularly interspaced palindromic repeats-associated protein 9 (CRISPR-Cas9) technology being utilized to enhance TIL, TCR-T, and CAR-T cell function by knocking out genes such as PCDCD1 to improve persistence, reduce exhaustion, and ultimately improve the outcomes in aggressive tumors such as PDAC [117,174,175,176,177].

In conclusion, expanding the therapeutic landscape of ACT through novel combinations and technological advancements holds promise for improving clinical outcomes. Continued research and collaboration will be critical in translating these findings into effective treatment options for PDAC and other challenging malignancies.

## 5. Conclusions

ACTs offer a promising approach for improving outcomes in PDAC, a malignancy with limited treatment options and poor prognosis. Despite significant challenges, ongoing advancements in immunotherapy, including CAR-T, TCR-T, TIL, CAR-NK, and CIK cell therapies, are paving the way for more effective and personalized treatment strategies. Overcoming barriers, such as immunosuppressive TME, target antigen selection, and therapy-related toxicities, is crucial for maximizing the potential of these therapies. Future research efforts should focus on optimizing the manufacturing processes, enhancing therapeutic efficacy through combination strategies, and fostering collaborative efforts among stakeholders. Ultimately, continued innovation and clinical investigation will be key to translating these promising therapies into viable treatment options for PDAC and other malignancies.

## Figures and Tables

**Table 1:** Current evidence of CAR-T cell therapies in pancreatic ductal adenocarcinoma.

Trial	Target	Outcomes	Adverse Effects	Notes on the Target
NCT02541370 * [52] (*n* = 23)	CD-133 (B) PDAC—7/23	PR—2 SD—3 PD—2	Hyperbilirubinemia, Anemia, Leucopenia, Thrombocytopenia, Anorexia, and Mucosal hyperemia	It is a transmembrane protein and the most commonly expressed cancer stem cell marker in several cancer types [53]. Correlates with histologic type, lymphatic invasion, and metastasis in pancreatic cancer [54].
NCT02850536 [55] (*n* = 5)	CEA	OS—23.2 m DOR—13 m	Fever, Electrolyte abnormalities, Hypertension	It can be elevated in PDAC and a level >7.2 ng/mL in LA-PDAC is often associated with systemic disease [56,57,58,59].
NCT01897415 [60] (*n* = 6)	Mesothelin	SD—2 PD—4	Abdominal pain, Back pain Dysgeusia, Gastritis	It is an important factor in pancreatic growth by promoting proliferation and inhibiting apoptosis through p53-dependent and p53-independent pathways [61,62]. Mesothelin-specific T cells were generated in 50% of pancreatic cancer patients in a study [63].
NCT02159716 [64] (*n* = 15)	Mesothelin (B) PDAC—5/15	PD—3 SD—2	Anemia, Lymphopenia, Fatigue, Dysgeusia, DIC	
NCT03874897 [65] (*n* = 37)	Claudin 18.2 (B) PDAC—5/37	PD—1 SD—3 PR—1	Lymphopenia, Neutropenia, Anemia, Thrombocytopenia, Elevated conjugated bilirubin, Elevated aminotransferase, Hypokalemia, Pyrexia	It is a transmembrane protein that controls the paracellular space through which molecules pass in the epithelial and endothelial tissues and is essential for normal membrane barrier function [46]. It is overexpressed in various cancers and plays an important role in the progression of pancreatic neoplasms. Claudin types could be tumor-specific.
NCT05199519 [66] (*n* = 7)	Claudin 18.2 (B) PDAC—2/5	PR—1 SD—1	Neutropenia, Anorexia	
NCT01869166 [67] (*n* = 14)	EGFR	PR—4 SD—8 PD—2	Lymphocytopenia, Pleural effusion, Pulmonary interstitial exudation, Dermatitis Herpetiformis, Gastrointestinal hemorrhage	It plays a crucial role in normal cellular growth, prevention of apoptosis, and development of metastasis in many types of cancer [68]. There are four receptors in the EGF family: HER1, HER2, HER3, and HER4 [69].
NCT01935843 [70] (*n* = 11)	HER2 (B) PDAC—2/11	SD—2	Anemia, Lymphopenia, Fever, Fatigue, Transaminase elevation, Gastrointestinal hemorrhage	It is a cell-membrane protein involved in promoting cell division and differentiation and contributes to tumor progression by triggering angiogenesis [71].

* Phase I/II; B—basket trials; PDAC—pancreatic ductal; adenocarcinoma; PR—partial response; SD—stable disease; PD—progressive disease; OS—overall survival; DOR—duration of response; CEA—carcinoembryonic antigen; EGFR—epidermal growth factor receptor; HER2—human epidermal growth factor receptor 2; DIC—disseminated intravascular coagulation.

**Table 2:** Ongoing CAR-T trials in pancreatic ductal adenocarcinoma.

Trial	Phase	Size	Target	Primary Outcome	Secondary Outcomes
NCT06464965	I	30	Claudin 18.2	MTD, DLT	ORR, DCR, DOR, PFS, OS
NCT05472857	I	30		MTD, AE	ORR, DCR, DOR, PFS
NCT04404595	Ib	110		MTD, DLT, AE, ORR	ORR, DCR, DOR, PFS, OS, HRQoL
NCT04581473	I/II	192		MTD, AE, PFS	ORR, DCR, DOR, PFS, OS
NCT05393986	I	63		MTD, DLT	ORR, DCR, DOR, PFS, OS, AE, PK
NCT05275062	I	6		AE	ORR, DCR, PFS, OS, CAR-T %, Tumor marker, RR, IM92 Ab
NCT06126406	I	60	CEA	DLT, AE	DCR, AUCS, CMAX, TMAX, CEA content
NCT06043466	I	30		MTD, DLT, Dose range	DCR, AUCS, CMAX, TMAX, CEA content
NCT06010862	I	36		MTD, AE	ORR, DCR, DOR, PFS, OS, AUCS, CMAX, TMAX, CEA content
NCT05736731	I/II	160		DLT, ORR, RP2D	A2B530%, Cytokine analysis
NCT04660929	I	48	HER 2	AE, Feasibility of manufacturing, CT—0508	ORR, PFS
NCT03740256	I	45		DLT	ORR, DCR, PFS, OS AEs grade 3
NCT06051695	I/II	230	Mesothelin	DLT, ORR, RP2D	A2B694 persistence, Cytokine analysis
NCT05239143	I	180	MUC1—C	MTD, ORR, R2PD	-
NCT06158139	I	27	B7-H3	AE, CRS, Neurotoxicity	ORR, DCR, PFS, OS, B7-H3 expression, DLT
NCT02830724	I/II	124	CD 70	AE within 2 weeks, RR	AE (within 6 weeks)

MTD—maximum tolerated dose; DLT—dose-dependent toxicity; AE—adverse events; CRS—cytokine release syndrome; RX—treatment; R2PD—recommended phase 2 dose; QOL—quality of life; ORR—objective response rate; CR—complete response; PR—partial response; DOR—duration of overall response; DOCR—duration of overall complete response; DCR—disease control rate; RRR—radiographic response rate; OS—overall survival; PFS—progression-free survival; RR—response rate (PR + CR); HRQoL—health-related quality of life; PK—pharmacokinetics; CEA—carcinoembryonic antigen; ACUS—area under the curve; CMAX—highest concentration of CEA CAR-T cells expanded; TMAX—time to reach the highest concentration.

**Table 3:** Ongoing adoptive cell therapy trials in pancreatic ductal adenocarcinoma.

	Trial	Phase	Size	Target	Outcomes
TIL therapy	NCT05098197	I	50	-	ORR, DCR, DOR, PFS, OS, TRAE
	NCT03935893	II	240	-	ORR, DCR, DOR, PFS, OS
	NCT04426669	I/II	20	-	MTD, PE, AE PFS, OS
	NCT01174121	II	332	-	ORR, TRAE Efficacy
	NCT05098197	I	50	-	ORR, DCR, DOR, PFS, OS, TRAE
CAR-NK	NCT03941457	I/II	9	ROBO1	TRAE
	NCT02839954	I/II	10	MUC1	TRAE ORR
	NCT03841110	I	64	NK cell + ICI	DLT ORR, DOR
	NCT06464965	I	30	Claudin18.2	MTD, DLT ORR, DCR, DOR, PFS, OS
	NCT05922930	I/II		TROP2	ORR, PFS, DLT
Cytokine-induced killer (CIK) cells	NCT03509298	II	90	CIK with anti-CD3-MUC1 bispecific antibody	ORR, DCR, PFS, OS, SSR, TTP
	NCT05955157	II/III randomized	52	DC-CIK_S-1 vs. S-1	TRAE, Hematological CBR Efficacy
T-cell receptor-engineered T-cells	NCT04809766	I	15	Mesothelin	TRAE ORR, PFS, OS
	NCT05438667	I	18	KRAS (G12V or G12D)	PFS, OS, AUC, CMAX, TMAX, AE, TTP, EFS, DFS, DoE TCR-T cell number, peak value of cytokines
	NCT06487377	I	12	KRAS (G12V or G12D)	DLT, TRAE, SAE ORR, DCR, DOR, PFS, OS, TTR, TCR-T cell counts, TCR gene copies, anti-drug antibodies, changes in tumor markers
	NCT04146298	I/II	30	KRAS (G12V)	ORR, TRAE OS, TCR transduced T cell %
	NCT06054984	I	18	RAS/TP 53	AUC, CMAX, TMAX, TRAE ORR, DCR, PFS, OS Change in tumor size, biomarker
	NCT06043713	I	24	KRAS (G12V)	MTD, DLT, AE ORR, PFS, OS, CBR, SD, changes in TME
	NCT05877599	I	162	TP53	ORR, DOR, PFS, DLT, AE, SAE, TRAE, CBR, TTR, BOR
	NCT06218914	I	24	KRAS (G12D)	DLT, AE, SAE ORR, DOR, PFS, OS, CBR, TTR, BOR
	NCT06105021	I/II	100	KRAS (G12V)	DLT, SAE, TEAE, OBD ORR, DOR, PFS, OS, CBR, TTR,
	NCT04622423	Observational	475		PFS, OS, tumor mutational burden, gene expression profile, antigenic landscape, T-cell repertoire, change in tumor marker
	NCT05964361	I/II	10	WT-1	DOR, BOR, leukapheresis %, SAE, ORR, DCR, PFS, OS, QoLA
	NCT03190941	I/II	110	KRAS (G12V)	TRAE, RR
	NCT03745326	I/II	70	KRAS (G12D)	TRAE, RR
	NCT04810910	I	20	Personalized Neo-antigen vaccine	OS, TRAE, RFS, CD4/CD8

TIL—tumor-infiltrating lymphocytes; CAR—chimeric antigen receptor; NK—natural killer cells; TRAE—treatment-related adverse events; ORR—objective response rate; CR—complete response; PR—partial response; DOR—duration of response; OS—overall survival; PFS—progression-free survival; MTD—maximum tolerated dose; PE—preliminary efficacy; LA—lab abnormalities; RR—response rate; DCR—disease control rate; QoLA—quality of life assessment; SIR—systemic immune response; DLT—dose-dependent toxicity; TTP—time to progression; SSR—symptom remission rate; CBR—clinical benefit rate; Cmax—peak plasma concentration; Tmax—peak time; AUC—area under concentration; BOR—best overall response.

## Data Availability

Not applicable.
